# Perspectives of push-pull-mooring effects on a desire for switching to alternative crops among tobacco farmers in Thailand: A qualitative study

**DOI:** 10.18332/tid/175685

**Published:** 2024-01-10

**Authors:** Chakkraphan Phetphum, Artittaya Wangwonsin, Atchara Prajongjeep, Saksin Simsin

**Affiliations:** 1 Department of Community Health, Faculty of Public Health, Naresuan University, Phitsanulok, Thailand; 2 Tobacco Control Research Unit, Naresuan University, Phitsanulok, Thailand; 3 Department of Community Public Health, Sirindhorn College of Public Health, Phitsanulok, Thailand

**Keywords:** tobacco farming, push-pull-mooring model, Thailand

## Abstract

**INTRODUCTION:**

Tobacco use contributes significantly to premature deaths worldwide. A key strategy to curb tobacco consumption involves limiting the tobacco supply through the transition to substitute crops. This study aims to provide insight into why tobacco farmers desire to switch to alternative crops and the support required for a successful transition.

**METHODS:**

Semi-structured interviews were conducted with 34 tobacco farmers expressing a desire to transition to other crops in two communities in Thailand. Data were recorded and transcribed verbatim in Thai and subsequently translated into English. A deductive content analysis applied the Push-Pull-Mooring (PPM) framework, contextualizing factors for the transition among tobacco farmers.

**RESULTS:**

Four main categories emerged: 1) push factors, encompassing negative experiences in growing tobacco, such as poverty, health problems, and hopelessness; 2) pull factors, representing positive experiences in transitioning to alternative crops, particularly having a role model; and 3) mooring factors, highlighting characteristic disadvantages of tobacco farmers, particularly the receipt of small tobacco growing quotas. The fourth category focused on the support needed for a successful transition, including the suspension of tobacco debt payments, access to low-interest loans, and the development of marketing capabilities.

**CONCLUSIONS:**

The study provides a comprehensive understanding of farmers' motivations to switch from tobacco to alternative crops and outlines the necessary support for a successful transition. Offering financial assistance and enhancing the production and marketing capabilities of alternative crops are essential steps toward facilitating a successful switch for farmers and ensuring a secure livelihood beyond tobacco farming.

## INTRODUCTION

The tobacco industry has long promoted the myth of tobacco growing as wealthy and economically prosperous, while evidence indicates that most tobacco farmers are impoverished, in debt, and live in poor conditions^1-5^. Those involved in tobacco production are likely to face higher risks of occupational injuries and health issues due to the toxins used in cultivation^6-11^, with some experiencing mental health problems linked to poverty and family tensions^12^. Providing concrete assistance to shift tobacco farmers away from cultivation and production is thus crucial, in accordance with Article 17 of the Framework Convention on Tobacco Control (WHO FCTC) which emphasizes the need to support alternatives to tobacco growing^13^.

Despite negative experiences and official efforts to promote alternatives, many tobacco farmers persist due to various crucial factors in crop selection for the upcoming season. A significant contributing factor to this persistence is the belief among farmers that tobacco is a high-profit and resilient crop with a stable market^14-16^. Furthermore, another key factor in the enduring commitment to tobacco cultivation is the loyalty demonstrated by farmers to the tobacco industry. This loyalty is strengthened by the support provided in the form of loans, rewards, and incentives, which serve as highly effective motivators^15,17^.

Thailand is a low-middle-income country that grows tobacco. Most tobacco leaf grown in the country supplies the local market, monopolized by the Tobacco Authority of Thailand under government supervision for setting quotas and producing cigarettes^17^. Tobacco purchase quotas are established by national committees, and cultivation follows contractual agreements, including a monopoly on seeds, fertilizers, and pesticides, with purchase prices influenced by quality inspections^17^. In 2022, cigarette sales revenue remitted to the government was about 39 billion THB (about 1.09 billion US$), with less than 1.1 billion THB (about 30.75 million US$) spent on purchasing tobacco leaves^18^.

In 2020, approximately 16000 tobacco farmers received the quotas for farming tobacco^18^. The common characteristics of tobacco farmers in Thailand typically include inheriting the practice from parents, having over 20 years of experience, being older, owning limited cultivation land, and often facing debt^5^. The economic issue is more serious, with a continuous decline in the amount of tobacco purchased by farmers in Thailand over the past six years resulting in a continuous decline in their incomes^18^. Recent research shows that reduced tobacco farming incomes correlate with lower societal, spiritual, and family quality of life among Thai tobacco farmers^5^. While this reason somehow plays a role in the desire of around half of the tobacco farmers in Thailand to switch from tobacco farming to alternative crops^6^, their specific motivations for doing so remain unclear.

While numerous studies address why tobacco farmers persist in cultivation^14,15,17^, there is a lack of evidence regarding motivations for ceasing tobacco farming. This study qualitatively explores the desires of some tobacco farmers to quit cultivation, emphasizing two research questions: 1) ‘Why do tobacco farmers want to switch to alternative crops?’, and 2) ‘What support is necessary for a successful transition?’.

To address the first question, this study employed the Push-Pull-Mooring (PPM) model^19,20^ originally designed for human migration. The model identifies ‘push’ and ‘pull’ influencing the desire to migrate, incorporating intrapersonal and lifestyle factors related to immigration^21^. The study adopted the PPM model, categorizing push factors as negative experiences in tobacco farming (original settlement) and pull factors as positive aspects of alternative crops (new settlement). Additionally, the study considered characteristic disadvantages influencing this transition and gathered farmers’ needs for government support in successfully transitioning from tobacco to alternative crops.

## METHODS

In this qualitative study, we examined the perspectives of tobacco farmers in Thailand, exploring their motivations to switch to tobacco alternatives and identifying the support needed for a successful transition. This method was chosen because it allows the collection of subjective information about interviewees’ thoughts, feelings, and beliefs on a particular subject, delving into personal perspectives^22^. This approach can handle sensitive subjects that may vary depending on the interviewee’s experience, perspective, and perception of the world^22^. The study received approval from the Committee in Human Research at Naresuan University. Furthermore, it did not receive funding from tobacco-related organizations or companies.

### Setting and participants

The study encompassed the geographical locales of Phrae Province and Sukhothai Province, recognized as principal tobacco-growing regions within Thailand^23^. Participants were purposively selected by Village Health Volunteers (VHVs) if they expressed interest in the interview and met the following eligibility: 1) interested in switching to alternative crops, and 2) being tobacco farmers in the past season. Snowball sampling was also employed to reach additional participants. Consequently, a total of 34 participants meeting the criteria participated in the study, comprising 25 tobacco farmers cultivating Virginian tobacco in Thung Si, Rong Kwang District, Phrae Province, and 9 farmers growing Burley tobacco in Thap Phueng, Si Samrong District, Sukhothai Province.

### Patient and public involvement

Members of the public and patients were not involved in the research design, analysis and dissemination.

### Procedures

The participants were first approached by VHVs of the community. We asked the VHVs to facilitate the creation of a list of those who agreed to participate, initially inform them about the purpose of the study, and make appointments for the interviews. The interview times were determined at the convenience of the interviewees, and all interviews took place in person at their houses to ensure a sense of privacy^22^. Semi-structured interviews were conducted between October 2021 and January 2022, by two experienced tobacco control researchers. The research team did not have any interaction with the participants before the interviews.

Prior to the interview, participants were provided with an information sheet detailing the study, informed consent forms outlining the confidentiality of their responses, as well as their right to participate or leave the interview without giving a reason. Permission for audio recording was also sought. The researchers conducted the interviews using a semi-structured interview guide^22^, developed by the research team and piloted with two random tobacco farmers. Two main questions were asked during the interviews: 1) ‘Why do you want to switch to growing alternative crops to tobacco?’, and 2) ‘What support do you want during the transition from tobacco to alternative crops?’. There was no language barrier between the participants and researchers, even though some spoke a local dialect (Northern Thai dialect). The responses were recorded using a recording application on the researchers’ smartphones, with each interview lasting around 90 minutes. An incentive of 300 THB was provided to participants after completing the interviews. Immediately after each interview, the audio files were sent to a researcher’s computer and securely stored in an encrypted folder, while the original files on the phones, were deleted.

### Data analysis

The research team members performed verbatim transcription of the interview recordings. A deductive qualitative analysis approach was employed to guide the data coding, emphasizing content analysis in accordance with the research framework^24^. Utilizing the Push-Pull-Mooring (PPM) model, codes and themes were generated as follows: 1) push effects, representing negative farming experiences leading to the consideration of switching to alternative crops; 2) pull effects, denoting positive factors of alternative crops replacing tobacco cultivation; 3) mooring effects, encapsulating personal limitations prompting tobacco farmers to switch to alternative crops; and 4) requirements, indicating the support needed for a successful transition. Following the content analysis guidelines of Saldaña^25^, one of the authors coded and categorized the interview transcriptions to ensure consistency across them. The codes and categories determined were then reviewed by other team members to verify their accuracy in reflecting participants’ perspectives. Subsequently, the results were translated into English.

## RESULTS

### Participants’ characteristics

A total of 34 tobacco farmers participated in this qualitative study. Of these, 29 individuals were males and 5 were females. The participant pool comprised 25 individuals who owned tobacco farms, while 9 participants worked within the tobacco farming sector. Among the participants, 26 were aged <60 years, with the remaining were aged ≥60. The age of participants ranged 35–71 years.

### Results of content analysis

Four categories were identified (Tables 1 and 2). The first three categories were generated in accordance with the PPM effects to address the first research question of why tobacco farmers want to switch to alternative crops to tobacco. These categories were supported by three sub-categories. The other, consisting of two sub-categories, was established to address the second question relevant to the support needed in transitioning to tobacco alternative crops.

**Table 1:** Categories and sub-categories based on research questions conducted among tobacco farmers in 2020, Phrae and Sukhothai Provinces, Thailand (N=37)

*Research questions*	*Categories*	*Sub-categories*
Why do tobacco farmers want to switch to alternative crops?	Push effects (negative factors for tobacco growing)	Poverty
		Health problems
		Hopelessness
	Pull effects (positive factors of tobacco alternative crop)	Having a role model
	Mooring effects (characteristic disadvantages of tobacco farmers)	Small quota received
What support do tobacco farmers need for a successful transition?	Requirements (support needed for a successful transition)	Suspending debt payments and accessing low-interest loans
		Developing marketing capabilities

**Table 2:** Representative quotes based on sub-categories by tobacco farmers in 2020, Phrae and Sukhothai Provinces, Thailand (N=37)

*Sub-category*	*Representative quotes*
Poverty	‘On this date, whatever you grow and earn money, you have to do. Growing tobacco cannot earn enough money, we have to change to grow something else. So, we must think about how much money is lost when the tobacco quota is cut. Will it be enough to spend all year round? No, not enough, so that need to find something else to grow … no one can stay still, all struggling almost to die.’
Health problems	‘Tobacco leaves have a strong smell. The leaf collectors often got a rash. I believe it’s from the leaves. They also contain toxins and pesticides.’
Hopelessness	‘Honestly, up until now, I have not seen an opportunity for tobacco to be as good as before. Tobacco farmers began to give up and lose hope.’
Having a role model	‘At first, I never thought that it would be possible to switch to other crops instead of tobacco. But when I had the opportunity to go and see work in other provinces where they grow chili and send it to a chili sauce factory. Farmers there earn more than when growing tobacco. So, I thought it might be possible to do it.’
Receiving small tobacco growing quotas	‘My problem is getting a quota less than 400. It’s difficult to continue. It’s impossible to cure. It’s a loss. If you want to do this, you have to make an effort, go to share the curing shed with someone else … the effect has come. Some farmers can’t endure and need to quit. There are less than 400 kg left. One shed has to put 8000–9000 kg. It’s not enough. It’s like forcing us to end up with stopping growing tobacco ...’
Suspending debt payments and accessing low-interest loans	‘Besides suspending debt payments, it would be good if they could make new loans with low interest so that we can invest in other crops without worrying. If they do not, farmers will have to get it from a loan shark, the problem will not be over.’
Developing marketing capabilities	‘For twenty years, I have only grown tobacco for my whole life. Want to plant, they bring seeds to us, finish planting and then go curing. After curing, go to sell it to the curing shed, whatever we earn we are all right, no need to think too much. But when you have to plant other plants, you have to think for yourself. Just growing it, anyone can do it, but what are you going to plant? (laughs) Who will you sell it for? How to set the price? I don’t know where the market is I only know about tobacco.’

### Push effects

Participants attributed negative experiences with tobacco farming to their desire to abandon the crop and switch to alternatives. The factors of push effects were categorized into three sub-categories: poverty, health problems, and hopelessness of tobacco cultivation.

*Poverty*

All 34 participants reported that the persistent reduction in tobacco leaf purchasing quotas for over five years significantly impacted the incomes of tobacco farmers and laborers. After deducting farming costs, many farmers experienced minimal profits or incurred capital losses, leading to insufficient income for living expenses and a reduced ability to pay debts. Consequently, some individuals acquired new debts, had to sell their farms, or migrated to other areas. This can be illustrated by one participant who stated that:

*‘Over the past ten years, our income had decreased a lot, depts had increased both old and new, some people had to sell their farms.’* (Participant 21)

and another said that:

*‘Some people had to move to work in another province.’* (Participant 3)

In addition, poverty also caused family problems. The main consequences were the quarrels in the family, demonstrated by one farmer that:

*‘We have more arguments in the family due to insufficient money.’* (Participant 8) and lack of money for a child’s education, as noted

by one participant that:

*‘My tears flowed when I called and told my child that I still had no money to send to her. I know that my child was financially suffering, but no, really no, felt embarrassed of the child and stressed about myself.’* (Participant 5)

All 34 participants cited the negative experiences as a result of poverty and debt as major reasons for switching to tobacco alternatives. As one participant expressed:

*‘On this date, whatever you grow and earn money, you have to do. Growing tobacco cannot earn enough money, we have to change to grow something else. So, we must think about how much money is lost when the tobacco quota is cut. Will it be enough to spend all year round? No, not enough, so that need to find something else to grow … no one can stay still, all struggling almost to die.’* (Participant 6)

*Health problems*

Three participants reported health problems resulting from the previous cultivation and harvesting of tobacco. This health issue served as a driving force to switch to a less impactful crop, with instances of short-term problems, such as a burning nose and itchy skin, attributed to exposure to toxins and chemicals from tobacco leaves:

*‘Tobacco leaves have a strong smell. The leaf collectors often got a rash. I believe it’s from the leaves. They also contain toxins and pesticides.’* (Participant 1)

Physical deterioration, resulting in musculoskeletal disorders, were viewed as cumulative long-term effects caused by working hard for long periods of time in repetitive postures as reflected by one farmer:

*‘Bend over and lift heavy in the sun all day. Doing it for thirty years, from young to old, now my legs are all weak, I think I really need to stop doing tobacco*.*’* (Participant 30)

Eight participants expressed that they had some health problems probably irrelevant to tobacco cultivation, but these health issues kept them from working hard on tobacco farms. This somehow prompted them to have an idea of switching to less labor-intensive alternative crops, such as fruit trees, which require a one-time investment but yield long-term benefits. In connection to this, one farmer stated that:

*‘I have both diabetes and high blood pressure. My children don’t want me to work hard. So, I want to switch to growing fruit trees such as tamarind and pomelo. It makes us tried once, but we can harvest and sell them for a long time ... it’s probably better than growing tobacco. It’s hard work. It has to be planted every year in the scorching sun.’* (Participant 12)

*Hopelessness*

Thirty key participants concurred that the consistent reduction in the tobacco purchasing quota by the Tobacco Authority of Thailand since 2017, currently with a 40–50% deduction compared to the highest quota received, directly resulted in lower incomes for tobacco farmers and laborers. Many farmers have acknowledged the unlikelihood of a resurgence in the tobacco farming industry, with some expressing desperation to continue growing tobacco:

*‘Honestly, up until now, I have not seen an opportunity for tobacco to be as good as before. Tobacco farmers began to give up and lose hope.’* (Participant 2)

Some people felt their pride in a career in tobacco farming diminished because they were so desperate for tobacco growing as indicated by this participant:

*‘My self-esteem has gone. What we have done since our parents’ generation that made us had a house and a car, now that’s not the case anymore*.*’* (Participant 28)

Acknowledging the prolonged reduction in the tobacco purchasing quota and the diminished potential for it to serve as a primary income source, some farmers, driven by desperation, are contemplating a shift to alternative crops as a newfound hope. This is reflected in the example of the interview:

*‘I have to accept that it is unlikely that I will get the same quota for growing tobacco back. It will continuously decrease. We have to only make up our minds. Now it is almost the end of this season. I think I will no longer grow tobacco I want to try planting something else*.*’* (Participant 13)

### Pull effects

A number of positive factors, attracting tobacco farmers to switch from tobacco to alternatives, were identified by the participants. These factors of pull effect were grouped into one sub-category, having a role model of crop replacement.

*Having a role model*

It was repeatedly reported by 20 participants that they had experience in a field trip and perceived the success of farmers who switched from tobacco to alternative crops. This experience enhanced their views on alternative opportunities for growing alternative crops to replace tobacco. As a result of this experience, they were able to view broader opportunities for growing alternative crops in place of tobacco as one said:

*‘At first, I have never thought that it would be possible to switch to other crops instead of tobacco. But when I had the opportunity to go and see work in other provinces where they grow chili and send it to a chili sauce factory. Farmers there earn more than when growing tobacco. So, I thought it might be possible to do that.’* (Participant 23)

As a result, the success of model farmers in switching from tobacco to alternative crops plays a significant role in influencing tobacco farmers to have an idea of discontinuing tobacco cultivation. Additionally, this increased confidence and willingness to experiment with planting alternative crops in place of tobacco, as indicated by this participant:

*‘For four or five years, I’ve thought about stopping growing tobacco, but I haven’t braved enough. When I noticed the communities that stopped growing tobacco and switched to asparagus, and their income improved, I could then decide that we could also succeed like them ... Now we have started to reduce tobacco planting areas to grow pumpkins and chilies instead, that is, let’s just subsist first. If it’s better, then we can expand the area.’* (Participant 11)

### Mooring effects

The participants reported a limitation in relation to their desire to switch to tobacco alternative crops. The factors of mooring effect were grouped into one subcategory which was a small quota received.

*Receiving small tobacco growing quotas*

According to two farmers, they wanted to switch to alternative crops because of their own limit caused by receiving a small tobacco growing quota. In other words, the quota was lower than the point of profitability. They viewed that small-scale farmers were receiving fewer quotas to the point where they would not be able to sustain their households. It is likely that tobacco farmers with the following characteristics would consider quitting tobacco cultivation in the near future. Based on their estimates, 400 kg are the minimum amount of quota to cover the capital of production per cycle. In the event that the quota is less than this, tobacco farmers will grow in less than a full ‘Rai’ (1 acre is about 2.5 Rai), which might result in losses from fertilizer or agricultural chemicals and labor costs. In connection to this, a farmer said:

*‘How to proceed? One rai can grow tobacco for at least 400 kilos. If we do not get enough quota, why would we do it? It’s not worth the fertilizer cost. Wages have not been counted yet*.*’* (Participant 19)

Another limitation of tobacco farmers who were allocated a small quota for growing tobacco was the cost of heating energy. Farmers would incur greater energy costs and risk greater losses if fewer tobacco leaves were to be cured than the curing plant’s capacity. Farmers whose tobacco growing quotas were reduced to less than 400 kg were likely to switch from tobacco to alternative crops as a result as indicated by one farmer that:

*‘My problem is getting a quota less than 400. It’s difficult to continue. It’s impossible to cure. It’s a loss. If you want to do this, you have to make an effort, go to share the curing shed with someone else … the effect has come. Some farmers can’t endure and need to quit. There are less than 400 kg left. One shed must put 8000–9000 kg. It’s not enough. It’s like forcing us to end up with stopping growing tobacco ...’* (Participant 2)

### Requirements

*Suspending debt payments and accessing low-interest loans*

Thirty farmers consistently stated that one of the most significant concrete supports for a successful transition from tobacco to alternatives was a long-term suspension of payment of tobacco-growing debts by the banks that were their creditors. Debts were accumulated from the purchase of fertilizers and agricultural chemicals, the construction of electric-powered tobacco curing ovens, and loans for tobacco cultivation, for example. This is illustrated by one farmer:

*‘Well, the debt accumulated from planting tobacco is very large and compounded continuously. If the bank agrees to suspend debt repayment for five or six years, it is still good when the money is received to invest in other livelihoods first*.*’* (Participant 12)

Furthermore, a total of 18 tobacco farmers repeatedly suggested that the banks should provide low-interest loans to tobacco farmers who have the intention to switch to alternative cash crops. For this reason, tobacco farmers could reinvest in other crops or alternative occupations in the long run as one farmer shared:

*‘Besides suspending debt payments, it would be good if they could make new loans with low interest so that we can invest in other crops without worrying. If they do not, farmers will have to get it from a loan shark, the problem will not be over.’* (Participant 30)

*Developing marketing capabilities*

A total of 13 participants commented that most tobacco farmers had received solid support and assistance from the Tobacco Authority of Thailand throughout their lives. This involved setting a planting quota and monopolizing seed cultivation as well as purchasing and determining prices. Growing alternative crops would therefore be their first experience with marketing. They generally lacked information and experience in dealing with marketing planning, such as selecting cash crops, and determining and bargaining fair prices. One farmer explained that:

*‘For twenty years, I have only grown tobacco for my whole life. Want to plant, they bring seeds to us, finish planting and then go curing. After curing, go to sell it to the curing shed, whatever we earn we are all right, no need to think too much. But when you have to plant other plants, you have to think for yourself. Just growing it, anyone can do it, but what are you going to plant? (laughs) Who will you sell it for? How to set the price? I don’t know where the market is I only know about tobacco.’* (Participant 2)

In addition, some had an idea to transform their agricultural products into products sold online such as chili paste and crackers, but they still lacked knowledge and skills about production standards (e.g. labels and packaging), and sales channels. In addition, marketing communications needed to be supported by relevant agencies. One farmer expressed:

*‘There are many households that want to make chili paste, and pumpkin crackers as a profession instead of growing tobacco. But it is stuck in that there is no knowledge of processed food production standards. If it is produced and will be sold online, what to do is still unknown. Therefore, I would like to have a coach to lead us to do it.’* (Participant 28)

## DISCUSSION

The present qualitative findings offer a comprehensive understanding of tobacco farmers’ motivations to shift to alternative crops. These reasons fall into a category of Push factors, encompassing adverse experiences such as poverty, health issues, and despair, and Pull factors, involving positive incentives like having a role model in the transition. Additionally, the desire to cease tobacco cultivation is influenced by mooring factors, particularly the constraint of receiving small tobacco growing quotas. Furthermore, the research underscores the necessity for supporting tobacco farmers in their transition by advocating for the suspension of tobacco debt payments, facilitating access to low-interest loans, and enhancing marketing capabilities.

Tobacco farmers in most tobacco-growing countries continue growing tobacco because they still view it as a cash crop that generates higher income than other crops^14,15^, and tobacco has outstanding plant features that allow it to endure harsh weather conditions, such as low rainfall and poor soil conditions, which are difficult to be replaced by other crops^15,16^. Moreover, a sense of loyalty to the tobacco industry has been fostered by the relationship values of ‘tobacco companies’ since farmers have received solid support over a period of time in the provision of credits as well as a stable tobacco market^16^, and incentives as well as cultivation materials^15,17^.

On the other hand, some tobacco farmers are considering quitting the crop. This qualitative research illustrated why tobacco farmers in Thailand want to stop growing tobacco and find a way to break the vicious cycle of tobacco cultivation and production. It was found that the ‘push’ factors that drive tobacco farmers to abandon tobacco cultivation, which is implied as their original settlement, to other alternative crops, which is implied as a new resettlement, was negative experiences of tobacco cultivation, such as poverty, health problems, and a feeling of despair toward tobacco cultivation. The ‘pull’ factor, a positive factor of alternative crops, was a result of seeing role models who were former tobacco farmers, and achieved stable income and a higher quality of life from alternative crops.

This study supports the finding of previous work indicating that the motivations for stopping tobacco growing included the belief that tobacco growing is not economically worthwhile and causes health problems and provides additional insight into the motivations for stopping tobacco growing. We found that the push effects related to negative experiences or lessons learned in tobacco cultivation were the most strongly identified factors that drove tobacco farmers to quit growing tobacco. Unsurprisingly, poverty was likely the strongest driving force that kept them from growing tobacco.

Although the tobacco industry has long propagated stories about the wealth and economic well-being of tobacco farmers, the findings of this study reinforce the empirical evidence from a number of previous studies^1-5^ asserting that most tobacco farmers are poor, in debt, and have a poor quality of life because of the tobacco farming cycle for most of their lives. Likewise, previous studies^2,26^ found that tobacco farmers in China and Uganda were willing to switch to alternative crops to tobacco when encountered decreased household income.

The effects, however, went beyond that. The findings from this study shed light on the depth that poverty is not only a lack of sufficient income to cover expenses but also a lack of opportunities in life. In the event of a debt crisis, for instance, agricultural land might have to be sold or farmers may have to migrate. As a result, they may lose both present and future opportunities and the potential of the capital and their household relationships. It may also affect their children’s chances of a good education, which is crucial to their future. Additionally, the long-term accumulation of poverty has reduced the hopes of tobacco farmers to the point where they have given up on a career in tobacco production. It is a significant impetus for tobacco farmers to seek new alternative crops.

Poor health also contributed to tobacco farmers’ decision to switch to other corps or occupations that may not have the same impact on their health as tobacco farming. Although a minority of the farmers believed and experienced health problems as a result of tobacco cultivation, well-established evidence supports that work related to tobacco cultivation has a high risk of adverse health effects both short-term (e.g. burning nose, itchy as a result of toxin and chemicals from tobacco) and long-term (e.g. physical deterioration and musculoskeletal problems as a result of long-term cumulative effects of working hard in repetitive positions), in line with several previous studies^6-11^.

Similar to the results of a study among tobacco farmers in Uganda^2^, it was found that perception of health impacts related to tobacco growing was one of the factors contributing to a desire to stop or keep distance from tobacco cultivation. In addition, when considering health as a significant input to farming, some tobacco farmers in Thailand believed that growing tobacco was not suitable for them since they were quite old and often had underlying illnesses such as chronic non-communicable diseases. Due to these limitations, they realized that they might not be able to work hard on tobacco farms anymore, which prompted them to switch to alternatives to tobacco that would require less labor and offer longer harvesting time.

On the other hand, participants’ perception of the lessons learned from former tobacco farmers, who had currently stable incomes and better quality of life, is the only factor of the pull effect for switching to alternative crops. In light of these findings, government agencies should consider providing current tobacco farmers with educational experiences of role or community models on how to successfully quit growing tobacco. This is in line with China’s successful model for promoting alternative crops^27^, which targeted tobacco farmers with a willingness to permanently stop growing tobacco. This approach was initiated by presenting a model community that has succeeded in quitting tobacco growing for tobacco farmers, as the first step in eight main activities.

This sort of activity should be designed to target both tobacco farmers without a desire for tobacco farming and those with such a desire but lack inspiration, in order to increase the positive pull effect to motivate tobacco farmers to explore possible alternatives to their economic livelihoods. Alternatively, it can consider those who are most likely to be affected by the reduction of the tobacco planting quota. In connection with this, this study found that small-scale tobacco farmers allocated less than 400 kg quota are unlikely to earn from the farms enough to sustain their households. Considering Virginia tobacco, the selling price is 95.24 THB per kg, with an average cost of 44.73 THB per kg, resulting in a net income of 20204 THB from a 400 kg yield^23^. For Burley tobacco, the selling price is 69.35 TH per kg, and the average cost is 28.50 TH per kg, leading to a net income of 16340 THB from a 400 kg yield^23^. If the yield for both varieties is less than 400 kg, falling below the provincial minimum daily wages of 308–330 THB^28^, farmers with this limitation may be compelled to quit the farm and are particularly vulnerable, necessitating support.

Regarding the support needed by the farmers for the successful transition, it seems that they have two main concerns: suspension of debt payment and accessibility to new loans. These issues are consistent with the evidence in the Philippines and Indonesia, middle-income and tobacco-growing countries, where financial capital and loan accessibility were crucial barriers to switching to tobacco alternatives^14^. There is a possibility that this is because tobacco farmers have long been accustomed to close patronage from the tobacco industry, both in terms of credit and support for their growing operations^15,17^. It is reasonable to assume that this is one strategy used to maintain tobacco farmers’ faithfully and continuously growing tobacco.

To oppose tobacco control and taxation measures, tobacco companies commonly assert the negative macroeconomic consequences to the nation and emphasize the impact on the quality of life for financially vulnerable tobacco farmers^14^. In relation to these circumstances, the government should pursue proactive measures to push for guidelines on concrete financial assistance. The use of this approach may encourage tobacco farmers to switch to alternative crops as an alternative to tobacco farming with a minimal impact^6^. Increasing access to credit and capital is fundamental to promoting sustainable agricultural development and farmers’ livelihood in low-income countries^29^.

Additionally, tobacco farmers wishing to switch to alternative crops frequently express concern about the lack of an available market for new crops^14^. In the present study, those who expressed that they were unwilling to grow other crops than tobacco was because they lacked the ability to market the new products. Several Thai farmers were concerned about the lack of access to information and lack of experience in market planning, such as the types of plants the market requires, the source of purchase, and the determination of fair prices. Therefore, good training in market capabilities would increase decision-making and increase the success of switching to alternative crops^26,30^. Particularly, this would facilitate older and less educated farmers to transition to other choices of making a living^31^.

### Limitations

This study has some limitations. Firstly, despite the fact that all interviewers were well versed in semi-structured interviews and the interviews were facilitated by VHVs as gatekeepers, it is possible that some participants did not express their actual opinions due to language limitations in their native tongue as well as confidentiality concerns. Secondly, the use of purposive and snowball sampling conducted within the narrow communities and focused on those interested in transitioning to alternative crops and meeting specific criteria, restricts the generalizability of findings to countries with different contexts. The findings have limited generalizability to other settings with different contexts. Thirdly, while semi-structured interviews provide a comprehensive understanding of how an individual feels and views a particular topic, it is possible that some perspectives may not be sufficiently comprehensive and generalizable to other contexts, since these interviews were conducted within the same communities. Lastly, local dialect to English translations may have obscured some meanings of the responses. The researchers minimized errors by checking all perspectives comprehensively.

## CONCLUSIONS

Tobacco cultivation and farmers have been strategically employed by the tobacco industry to impede the progress of tobacco control. While a notable number of smallholder tobacco farmers in Thailand express a desire to cease tobacco cultivation and transition to alternative crops, they often lack the confidence to make such a shift. This study delved into the push, pull, and mooring effects influencing this desire and identified the necessary support for a successful transition. The study revealed that negative experiences in tobacco farming, such as poverty, poor health, and a sense of hopelessness, served as push factors compelling farmers to contemplate abandoning the tobacco crop. Conversely, the presence of a role model who had successfully transitioned to alternative crops emerged as a pulling force, fostering farmers’ willingness to switch. Furthermore, a characteristic disadvantage among farmers, namely receiving a small tobacco planting quota, contributed significantly to their desire to transition to a different crop. In addition to exploring these factors, the study identified the crucial support farmers require for a successful transition, including the suspension of debt payments and access to low-interest loans. The findings suggest that offering financial assistance and enhancing the production and marketing capabilities of alternative crops could facilitate a successful switch for farmers, ensuring a secure livelihood beyond tobacco farming.

## Data Availability

The data supporting this research are available from the authors on reasonable request.
